# Identification and validation of the necroptosis-related gene signature related to prognosis and tumor immune in hepatocellular carcinoma

**DOI:** 10.1097/MD.0000000000030219

**Published:** 2022-09-09

**Authors:** Zhiping Xiang, Geofrey Mahiki Mranda, Xingguo Zhou, Ying Xue, Yu Wang, Tian Wei, Junjian Liu, Yinlu Ding

**Affiliations:** a Department of Gastrointestinal Surgery, The Second Hospital, Cheeloo College of Medicine, Shandong University, Jinan, Shandong, China.

**Keywords:** gene signature, hepatocellular carcinoma, immune infiltration, necroptosis, overall survival

## Abstract

Hepatocellular carcinoma (HCC) is the sixth most common cancer, which is characterized by complicated etiology, excessive heterogeneity, and poor prognosis. Necroptosis is a new kind of programmed cell death, which is intently associated with the occurrence and development of tumors. Although researchers have had a deep understanding of necroptosis in recent years, the expression level of necroptosis-related genes in HCC and its relationship with the survival time of HCC patients are not clear.

**Methods:**

According to the expression of necroptosis-related genes and the survival of HCC patients, HCC patients in the TCGA database were divided into 2 groups that were relatively independent of each other. The genes related to the survival time of HCC patients were screened from the 2 groups of differentially expressed genes. By using the Least Absolute Shrinkage and Selection Operator Cox regression analysis, the optimal λ value was obtained, and the 10-gene signature model was established.

**Results:**

According to the median risk score of the TCGA cohort, HCC patients were averagely divided into high- and low-risk groups. Compared with the low-risk group, the death toll of the high-risk group was relatively higher and the survival time was relatively shorter. Principal component analysis and t-distributed stochastic neighbor embedding analysis showed that there was a significant separation between high- and low-risk groups. Through Kaplan–Meier analysis, it was found that the survival time of HCC patients in the high-risk group was significantly shorter than that in the low-risk group. Through receiver operating characteristic analysis, it was found that the sensitivity and specificity of the model were good. We also make a comprehensive analysis of the international cancer genome consortium database as a verification queue and prove the reliability of the 10-gene signature model. Gene Ontolog, Kyoto Encyclopedia of Genes and Genomes, and single-sample gene set enrichment analysis showed that many biological processes and pathways related to immunity had been enriched, and the antitumor immune function was weakened in the high-risk population.

**Conclusion:**

The risk score can be considered as an independent prognostic factor to predict the prognosis of patients with HCC, and necroptosis-related genes are also closely related to tumor immune function.

## 1. Introduction

Hepatocellular Carcinoma (HCC) is the third leading cause of cancer death worldwide in 2020, with approximately 906,000 new cases and 830,000 deaths, and liver cancer ranks fifth in terms of global incidence and second in terms of mortality for men.^[1]^ HCC is a kind of cancer with obvious heterogeneity among patients, between tumors and within tumors.^[2–4]^ The main risk factors for HCC are chronic infection with hepatitis B virus or hepatitis C virus, aflatoxin-contaminated foods, heavy alcohol intake, excess body weight, type 2 diabetes, and smoking.^[1]^ The complexity of the etiology of liver cancer and the high heterogeneity of liver cancer make liver cancer the third largest cause of cancer death in the world. The recent progress in the treatment of liver cancer is remarkable, and targeted therapy enriches the treatment of HCC and breaks the dilemma of HCC treatment to some extent. With the help of the clinical prediction model, doctors and patients can better make common decisions, clinical researchers can screen suitable research objects more accurately, and government departments and health managers can better allocate medical resources. The prognostic model study is to establish, verify and evaluate the statistical model to predict the future outcome of patients. The prognostic model can provide an objective prediction of the risk of events and can be used as a supplement to clinicians’ subjective judgment and clinical diagnosis and treatment guidelines. Accurate prediction results can improve the clinical decision-making ability of doctors and thus improve the prognosis of patients. The establishment of a prognostic model can better predict the prognosis and reveal the gene therapeutic targets related to prognosis, so a reliable prognostic model is very necessary.

**Figure 1.**
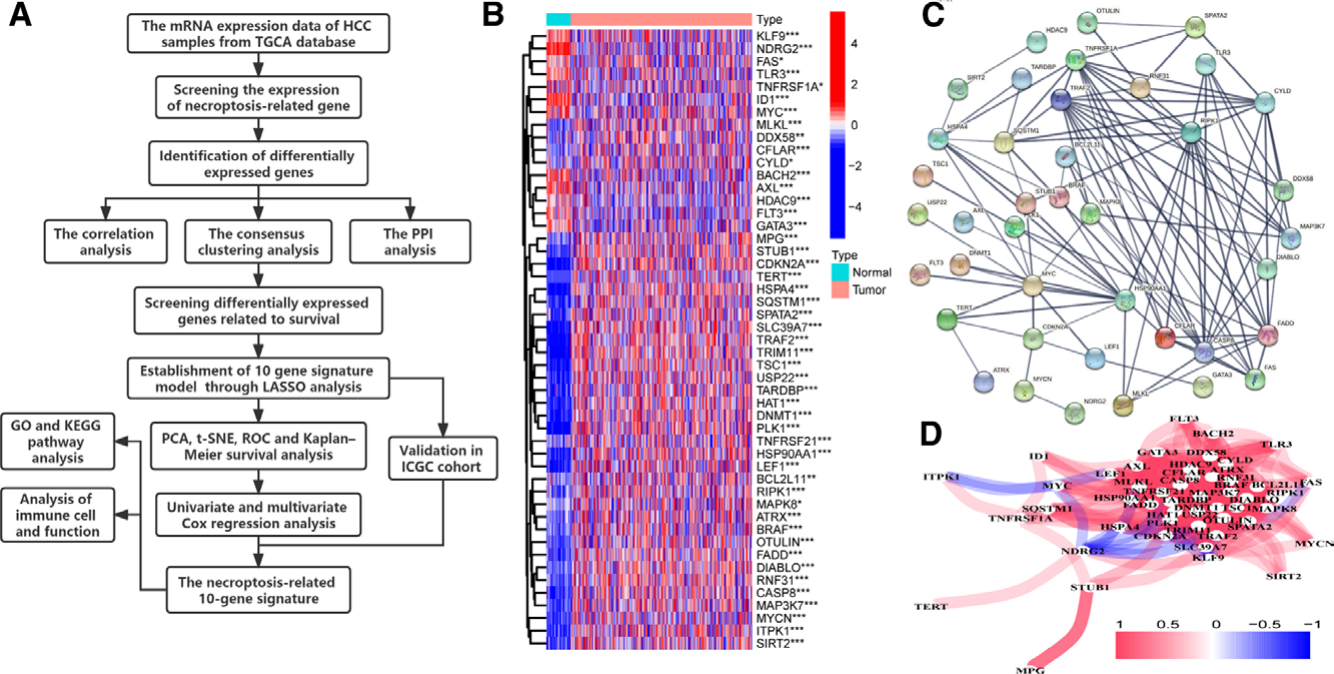
(A) Flow chart of this research. The expression level of 49 necroptosis-related genes and the relationship between them. (B) The necroptosis-related DEGs heatmap of normal and HCC tissues in the TCGA cohort (*P value < .05, **P value < .01, ***P value < .001). (C) The PPI network shows the interactions of the necroptosis-related DEGs. (D) The related network of necroptosis-related DEGs. DEG = differentially expressed gene, PPI = protein–protein interaction.

Necroptosis, as a novel programmed form of cell death different from apoptosis, can enhance CD8 + leukocyte-mediated antitumor immunity by activating RIPK1 and RIPK3 within the tumor microenvironment.^[5]^ As the study of necroptosis continues, researchers have found that necroptosis is closely related to the progression of cancer. At the same time, necroptosis plays an important regulatory role in the occurrence, development and metastasis of cancer.^[6–9]^ The researchers found that necroptosis is associated with antitumor immunity, which promotes tumor metastasis and T cell death.^[10–13]^ Although researchers have made a lot of discoveries about the mechanisms of necroptosis, the specific role and potential molecular mechanism of necroptosis in the prognosis of HCC are not clear. It is unclear whether these necroptosis-related genes are associated with the prognosis of patients with HCC. In this article, we conducted a very targeted study to understand the prognostic value of necroptosis-related genes. In addition, we studied the relationship between necroptosis-related genes and tumor immune microenvironment. The flow chart of this research is shown in Figure 1A.

## 2. Materials and Methods

### 2.1. Data collection

Download the RNA sequencing (RNA-seq) data and the corresponding clinical survival data about Hepatocellular Carcinoma from TCGA database (https://portal.gdc.cancer.gov). There are 424 samples, including 50 normal samples and 374 GC samples. The RNA-seq data and clinical information of the external validation cohort were downloaded from the international cancer genome consortium (ICGC) portal (https://dcc.icgc.org/projects/LIRI-JP). Standardize the data collected from the database. The 68 necroptosis-related genes were found from related kinds of literature and are presented in (Table S1, Supplemental Digital Content, http://links.lww.com/MD/H96).

### 2.2. Construction and validation of the prognostic necroptosis-related gene signature

The “limma” software package was used to identify necroptosis-related differentially expressed genes (DEGs) between normal samples and HCC samples by R language version 4.1.1. The protein–protein interaction (PPI) network of DEGs was constructed with the Search Tool for the Retrieval of Interacting Genes (STRING; https://string-db.org/). We set the minimum interaction score for constructing a PPI network to 0.7 (high confidence). Using R language to construct the correlation network of necroptosis-related DEGs, and the cutoff value was 0.2.

Through consensus clustering analysis, the 370 patients with HCC in the TCGA cohort were divided into several independent groups. In order to divide 370 patients with HCC into several clusters with high correlation within groups and low correlation between groups, we continuously adjust the value of the clustering variable (K) until the most satisfactory results were found. The necroptosis-related DEGs in the clusters were screened, and the clinical features and overall survival (OS) time of the clusters were compared.

The prognosis-related genes were screened by univariate Cox regression analysis from DEGs in the clusters. Then, the prognosis-related DEGs were analyzed by the Least Absolute Shrinkage and Selection Operator (LASSO) Cox regression analysis with the “glmnet” software package of R language version 4.1.1. Finally, the necroptosis-related gene signature model was constructed according to the optimum penalty parameter (λ) value. The risk score formula was as follows: risk score = ∑ Xi*Yi (X: coefficient, Y: gene expression level). The risk score was calculated after the standardization of TCGA expression data using the “scale” function of R language version 4.1.1. The 370 HCC patients could be divided into high- and low-risk groups by the median of the risk score of the TCGA cohort. The information of 240 patients with HCC was obtained by ICGC (LIRI-JP) and was used as validation sets to validate the gene model. In order to facilitate the research, the “scale” function was used to normalize the gene expression data. The risk score was calculated using the same formula as the TCGA cohort. According to the median risk score in the TCGA cohort, 240 patients in the ICGC cohort were divided into high- and low-risk groups. The principal component analysis (PCA), t-distributed stochastic neighbor embedding (t-SNE) analysis, and Kaplan–Meier survival analysis were respectively performed between the high- and low-risk groups of the TGCA and ICGC cohort. The sensitivity and specificity of the model were evaluated by time-dependent receiver operating characteristic (ROC) analysis.

We extracted the clinical information of patients in the TCGA cohort and the ICGC cohort. In our regression model, these clinical variables and risk scores in combination were analyzed. Whether the risk score can independently predict the prognosis of patients with HCC needs further research. Therefore, we verify this problem by univariate and multivariate Cox regression analysis. In addition, the clinical features heatmap of the TCGA cohort was generated to find out whether the clinical characteristics of patients were different between the high- and low-risk groups.

### 2.3. Functional enrichment analysis and comparison of the immune activity between subgroups

We used the “limma” software package of R language version 4.1.1 to extract the DEGs of the TCGA cohort from high- and low-risk groups (|log2FC|≥1, false discovery rate < 0.05) and carried out Gene Ontology (GO) enrichment analysis and Kyoto Encyclopedia of Genes and Genomes (KEGG) pathway analysis of DEGs from high- and low-risk groups to explore the differences of gene functions and pathways among the high- and low-risk groups by the “cluster Profiler” package of R language version 4.1.1.

In order to further explore the enrichment scores of 16 kinds of immune cells and the activities of 13 immune-related pathways in high- and low-risk groups of the TCGA cohort, we carried out the single-sample gene set enrichment analysis (ssGSEA) by “gsva” package of R language version 4.1.1.

## 3. Results

### 3.1. Identification of necroptosis-related DEGs between normal and HCC tissues

By comparing the expression levels of 68 necroptosis-related genes in 50 normal tissues and 374 HCC tissues in the TCGA database, 49 DEGs were identified (*P* value < .05). Eleven genes (ID1, BACH2, NDRG2, AXL, MYC, GATA3, TLR3, FLT3, KLF9, TNFRSF1A, and FAS) were downregulated in HCC samples. The other 38 genes (HDAC9, DDX58, CYLD, MAPK8, CFLAR, MPG, RIPK1, TARDBP, SIRT2, BRAF, BCL2L11, OTULIN, ATRX, DIABLO, STUB1, SPATA2, MLKL, MAP3K7, HAT1, FADD, ITPK1, CASP8, HSP90AA1, RNF31, HSPA4, USP22, TNFRSF21, SLC39A7, TSC1, SQSTM1, TRIM11, TRAF2, DNMT1, LEF1, PLK1, MYCN, CDKN2A, and CDKN2A) were upregulated in HCC samples (Fig. 1B). The PPI analysis can help us to further understand the interactions between necroptosis-related DEGs between the normal and HCC samples (Fig. 1C). The minimum interaction score of the PPI analysis was set at 0.7 (high confidence). The correlation network of necroptosis-related DEGs between the normal and tumor samples obtained by R language is shown in Figure 1D.

### 3.2. Tumor classification based on necroptosis-related genes

**Figure 2.**
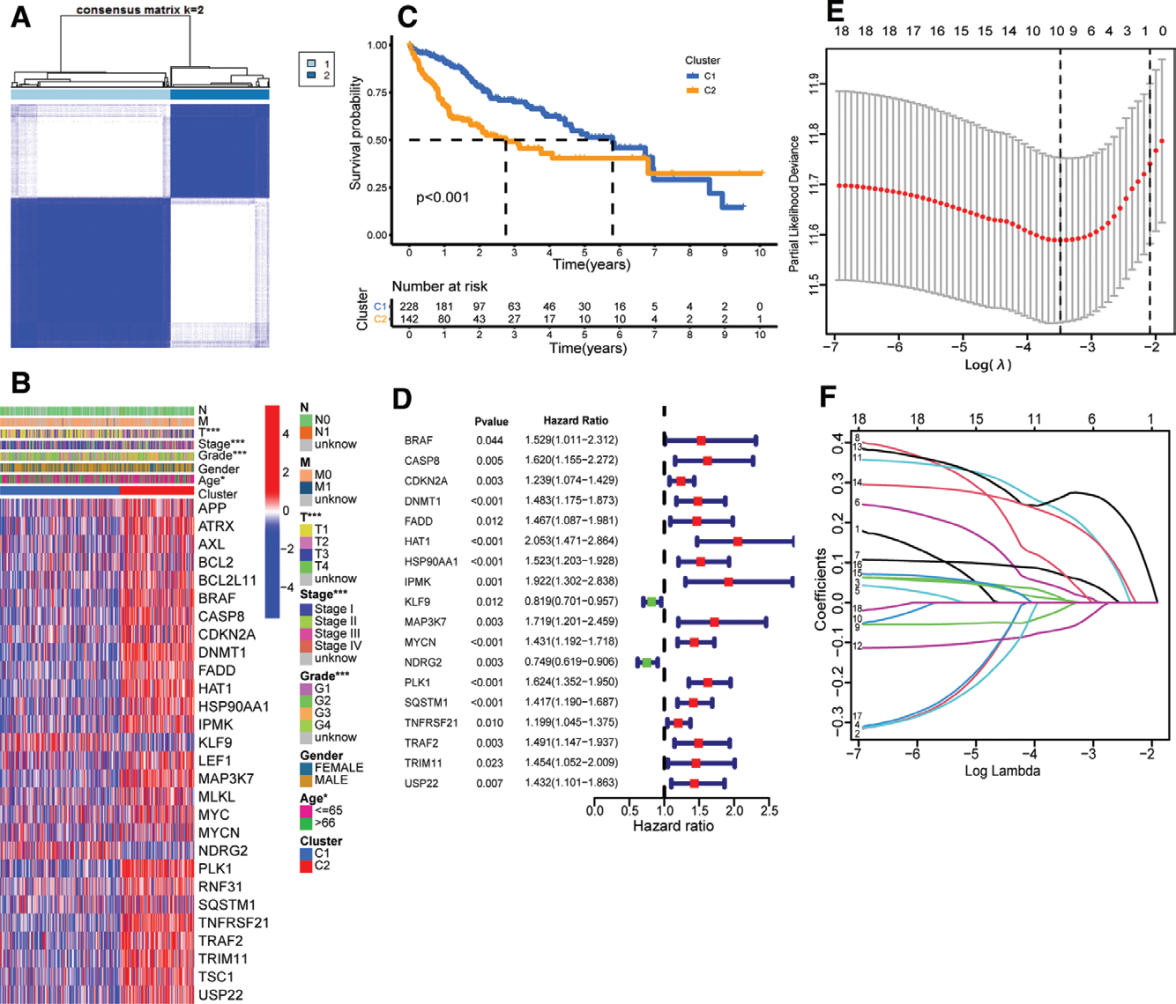
Classification and risk signature model of HCC samples in the TCGA cohort. (A) The patients with HCC were divided into 2 clusters according to the consensus clustering analysis. (B) heat map of clinical characteristics of patients and DEGs in the 2 clusters generated by consensus clustering analysis (*P value < .05, **P value < .01, ***P value < .001). (C) The Kaplan–Meier survival analysis of 2 clusters generated by the consensus clustering analysis. (D) Eighteen genes related to the prognosis of HCC patients were screened by the univariate Cox regression analysis. (E) The cross-validation analysis of parameter in LASSO regression analysis of 10 necroptosis-related genes. (F) The LASSO regression analysis of 10 necroptosis-related genes. DEG = differentially expressed gene, HCC = hepatocellular carcinoma, LASSO = Least Absolute Shrinkage and Selection Operator.

We conducted the consensus clustering analysis on all 370 patients with hepatocellular carcinoma in the TCGA cohort to explore the relationship between the expression level of 68 necroptosis-related genes and HCC subtypes. In order to obtain the groups with a high correlation within the group and the low correlation between groups, we constantly adjust the clustering variable (k) and find that when k = 2, the grouping effect is the best. The 370 patients with HCC can be well divided into 2 clusters according to 68 necroptosis-related genes (Fig. 2A).

The heat map showed the gene expression profile and clinical features (Fig. 2B), including age (≤60 or >60 years old), grade (G1–G3), stage (I, II, II, III, V), and gender (male or female). We found that there were significant differences in HCC grade and stage between the 2 groups (*P* value < .001), and there were also gender differences between the 2 groups (*P* value < .05). Then, the OS of the 2 groups was compared, and there was a significant difference between the 2 groups (*P* value < .001; Fig. 2C). The analysis proved that we successfully divided the patients into 2 relatively independent clusters by consensus clustering analysis.

### 3.3. Construction and validation of the prognostic gene mode

**Figure 3.**
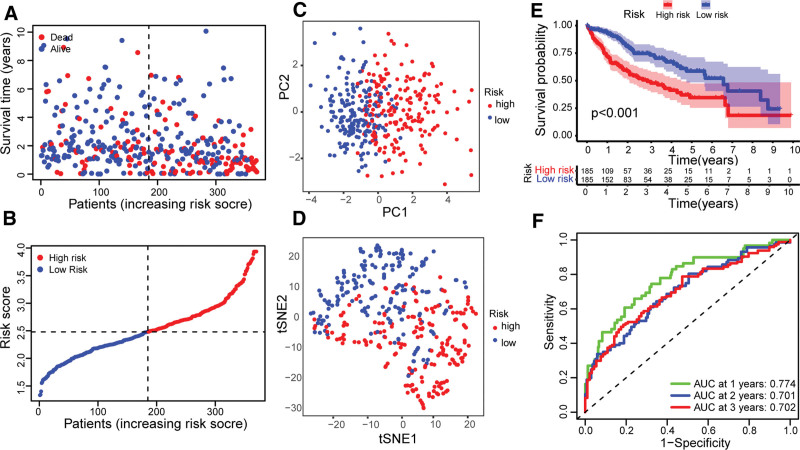
Construction of risk signatures model in TCGA cohort. The survival status of patients of high- and low-risk groups in (A) TCGA cohorts based on risk score. The distribution of patients of high- and low-risk groups in (B) TCGA cohorts based on the risk score. PCA analysis of high- and low-risk groups in (C) TCGA cohorts. t-SNE analysis of high- and low-risk groups in (D) TCGA cohorts. Kaplan–Meier survival analysis of high- and low-risk groups in (E) TCGA cohorts. ROC analysis of high- and low-risk groups in (F) TCGA cohorts. PCA = principal component analysis, ROC = receiver operating characteristic, t-SNE = t-distributed stochastic neighbor embedding.

**Figure 4.**
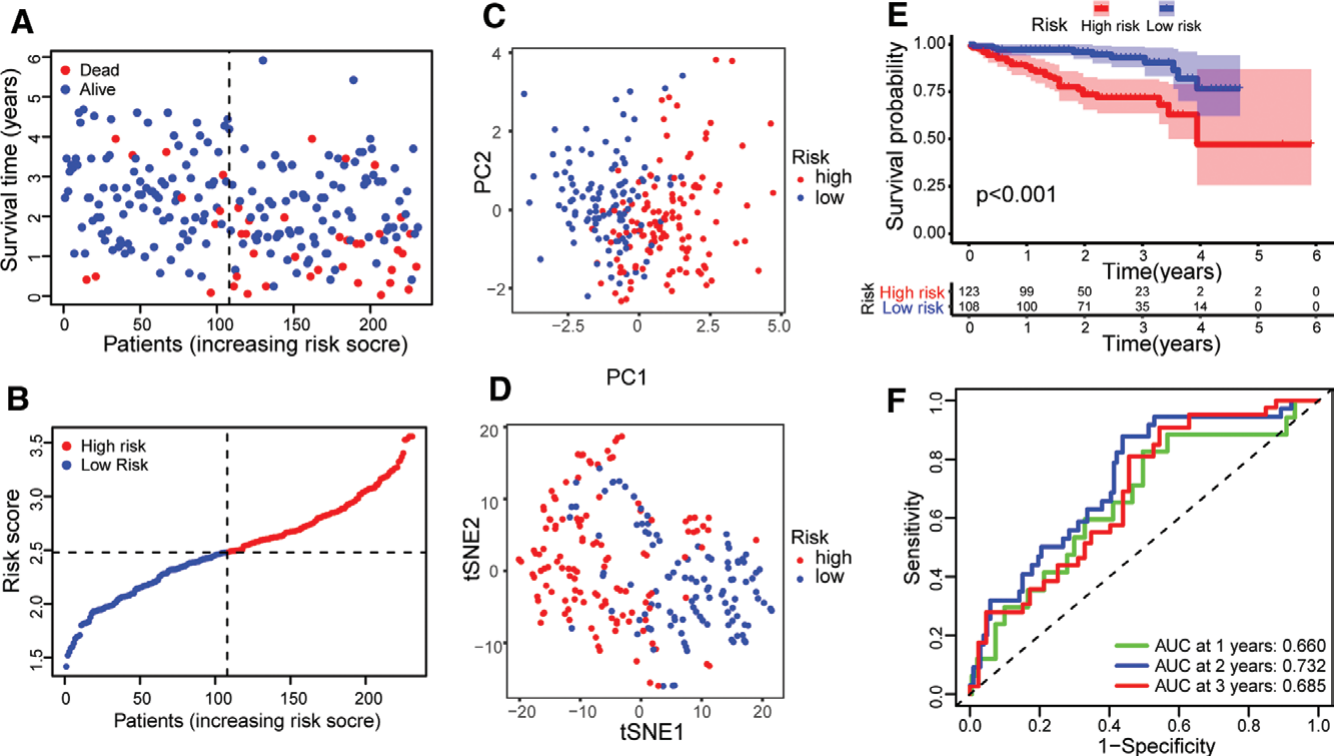
Verification of risk signatures model in ICGC cohort. The survival status of patients of high- and low-risk groups in (A) ICGC cohorts based on risk score. The distribution of patients of high- and low-risk groups in (B) ICGC cohorts based on the risk score. PCA analysis of high- and low-risk groups in (C) ICGC cohorts. t-SNE analysis of high- and low-risk groups in (D) ICGC cohorts. Kaplan–Meier survival analysis of high- and low-risk groups in (E) ICGC cohorts. ROC analysis of high- and low-risk groups in (F) ICGC cohorts. ICGC = international cancer genome consortium, PCA = principal component analysis, t-SNE = t-distributed stochastic neighbor embedding.

Through screening, 370 HCC samples in the TCGA cohort with complete survival information of patients were obtained. The genes related to the survival time of HCC patients were screened by univariate Cox regression analysis. Through analysis, The 18 genes related to the survival time of HCC patients were obtained (*P* value < .05, Table S2, Supplemental Digital Content, http://links.lww.com/MD/H97). We found that 2 genes (KLF9 and NDRG2) are hazard ratios (HRs) < 1, which are low-risk genes in the development of HCC. The HR values of the other 16 genes were all >1, which indicated that they were high-risk genes (Fig. 2D). The LASSO Cox regression analysis was used to obtain the optimal λ value (Fig. 2E, F). Then, the 10-gene signature model was constructed according to the optimum λ value. The risk score was calculated as follows: risk score = (0.007 * CDKN2A exp.) + (0.032 * HAT1 exp.) + (0.067 * HSP90AA1 exp.) + (0.069 * IPMK exp.) + (–0.013 * KLF9 exp.) + (0.224 * MYCN exp.) + (–0.064 * NDRG2 exp.) + (0.267 * PLK1 exp.) + (0.206 * SQSTM1 exp.) + (0.006 * TNFRSF21 exp.). According to the median risk score of the TCGA cohort, the 370 patients of HCC were averagely divided into high- and low-risk groups. Compared with the low-risk group, the number of deaths in the high-risk group was relatively higher, and the survival time was also relatively shorter (Fig. 3A, B). The PCA and t-SNE analysis showed that there was a clear separation between the high- and low-risk groups (Fig. 3C, D). Through Kaplan–Meier analysis, it was found that there was a significant difference in OS time between high- and low-risk groups (*P* value < .001; Fig. 3E). The survival time of patients in the high-risk group was significantly shorter than that in the low-risk group. The sensitivity and specificity of the model were analyzed and evaluated by time-dependent ROC. The ROC analysis of the TCGA cohort found that the areas under the curve of 1-, 2-, and 3-year survival were 0.774, 0.701, and 0.702 (Fig. 3F), respectively. A total of 231 HCC patients from the ICGC cohort were used as validation sets. According to the median risk score in the TCGA cohort, the HCC patients in the ICGC cohort were divided into high- and low-risk groups, and patients in the low-risk group had longer survival time and lower mortality than the high-risk group (Fig. 4A, B). PCA analysis and t-SNE analysis showed that there was a clear separation between the 2 groups (Fig. 4C, D). Kaplan–Meier analysis showed that there was a significant difference in survival rate between the low-risk group and the high-risk group (*P* value < .001; Fig. 4E). Compared with the high-risk group, patients in the low-risk group had a longer survival time. The ROC analysis of the ICGC cohort shows that the areas under the curve of 1-, 2-, and 3-year survival were 0.660, 0.732, and 0.685 (Fig. 4F), respectively.

### 3.4. The prognostic analysis of the risk score

**Figure 5.**
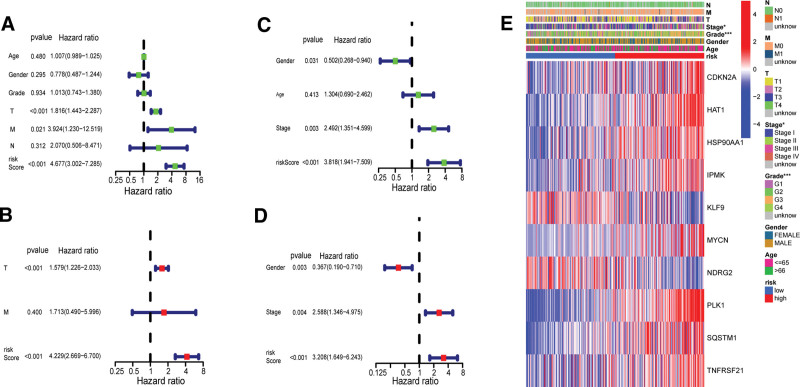
The univariate and multivariate Cox regression analyses of the risk score and clinical characteristics of patients in the TCGA cohort and the ICGC cohort. The univariate analysis was performed on the risk score and clinical characteristics of patients in the (A) TCGA cohort and the (C) ICGC cohort. The multivariate analysis was performed on the risk score and clinical characteristics of patients in the (B) TCGA cohort and the (d) ICGC cohort. (E) The heat map of DEGs and clinical characteristics of patients in high- and low-risk groups (*P value < .05, **P value < .01, ***P value < .001). DEG = differentially expressed gene, ICGC = international cancer genome consortium.

In order to verify that the risk score obtained by the 10-gene signature can be used as a good prognostic factor, we used univariate and multivariate Cox regression analysis to prove it. Through the univariate Cox regression analysis of the TCGA cohort, we found that the risk score was a predictor of the poor prognosis of HCC patients (Fig. 5A). Then, after adjusting for other confounding factors, the multivariate analysis also found that the risk score was an independent predictor of poor prognosis of HCC patients in the TCGA cohort (Fig. 5B). Confounding factors, also known as confounding factors or external factors, are related to research factors and diseases. If confounding factors are ignored, the real relationship between research factors and disease may be masked or exaggerated. In this study, age, grade, and stage are confounding factors, and ignoring these factors can lead to misestimation of the relationship between risk scores and prognosis of patients with liver cancer. We selected the ICGC cohort as the verification cohort, conducted the univariate Cox regression analysis and the multivariate analysis, and also found that the risk score was an independent predictor of poor prognosis of HCC patients in the ICGC cohort (Fig. 5C, D). Through the analysis, we have obtained the clinical characteristic heat map of the TCGA cohort and found that the grading and staging of patients were significantly different between high- and low-risk groups (*P* value < .05,;Fig. 5E).

### 3.5. Results of functional enrichment analysis and comparison of the immune activity between subgroup

**Figure 6.**
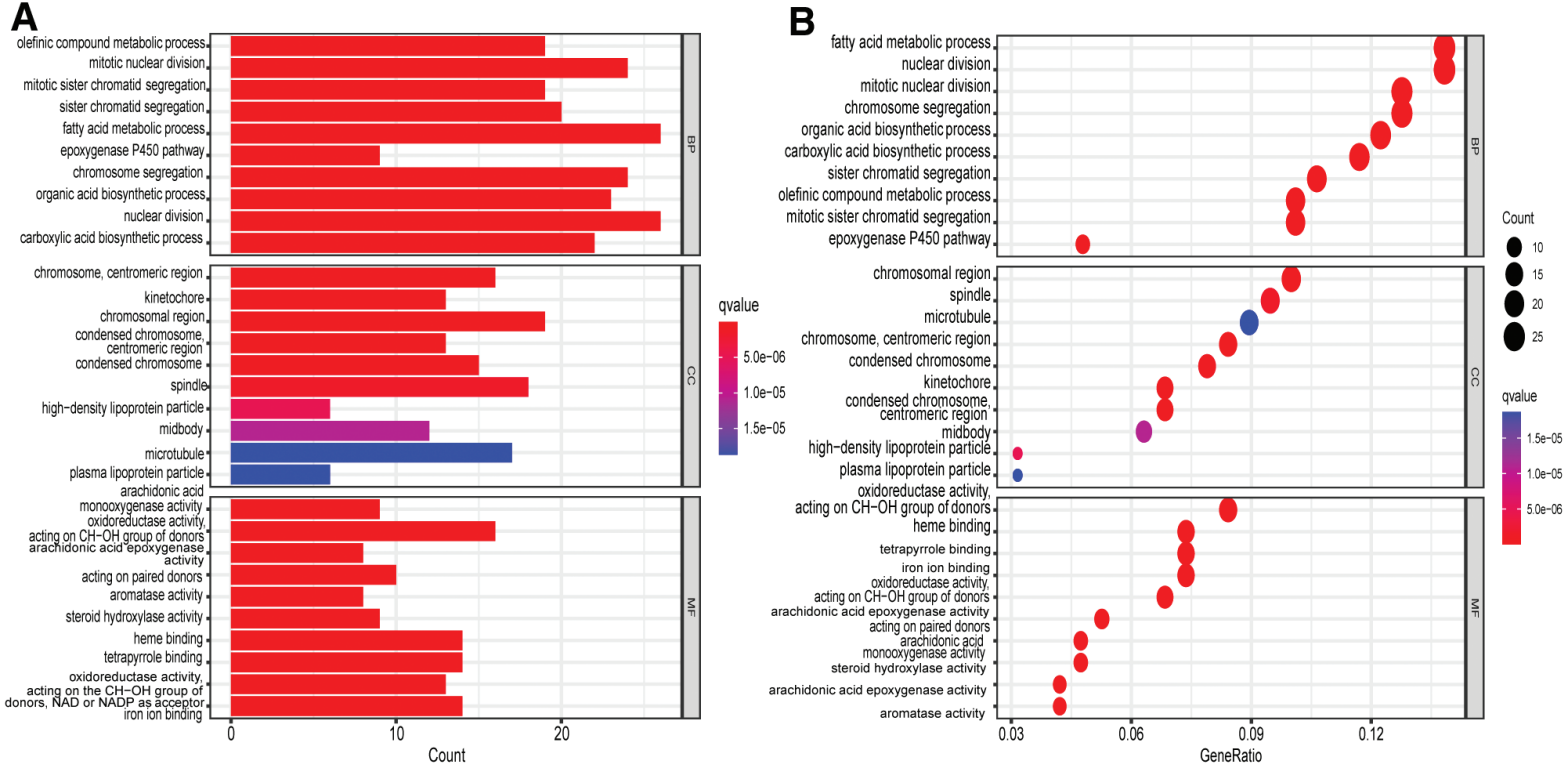
GO functional analysis of differentially expressed genes between high- and low-risk groups in the TCGA cohort. (A) GO barplot, (B) GO bubble graph. GO = Gene Ontology.

**Figure 7.**
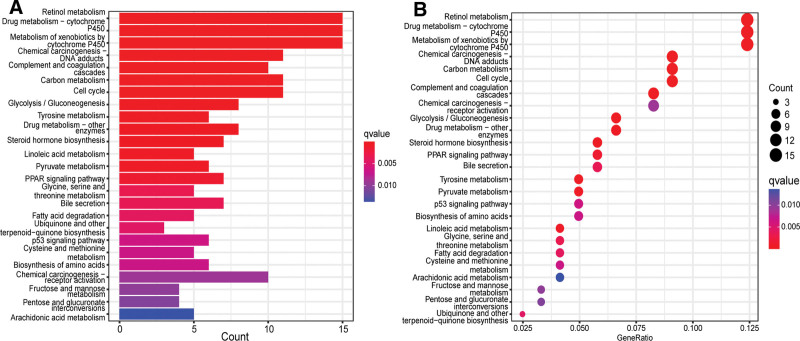
KEGG functional analysis of differentially expressed genes between high- and low-risk groups in the TCGA cohort. (A) GO barplot, (B) GO bubble graph. GO = Gene Ontology, KEGG = Kyoto Encyclopedia of Genes and Genomes.

We conducted a series of analyses to study the differences in gene functions and pathways between high- and low-risk groups. We used the “LIMMA” package of R language version 4.1.1 to extract DEGs between high- and low-risk groups in the ICGC cohort (false discovery rate < 0.05, |log2FC| ≥ 1). A total of 193 DEGs were identified between high- and low-risk groups in the TCGA cohort. Through the analysis of differential expression genes, we know that 97 genes were upregulated in the high-risk group and the other 96 genes were downregulated (Table S3, Supplemental Digital Content, http://links.lww.com/MD/H98). On this basis, GO enrichment analysis and KEGG pathway analysis were carried out. The results of functional enrichment analysis show that the necroptosis-related DEGs between high- and low-risk groups were related to fatty acid metabolic process, nuclear division, retinol metabolism, drug metabolism-cytochrome P450, and metabolism of xenobiotics by cytochrome P450 (Figs 6 and 7).

**Figure 8.**
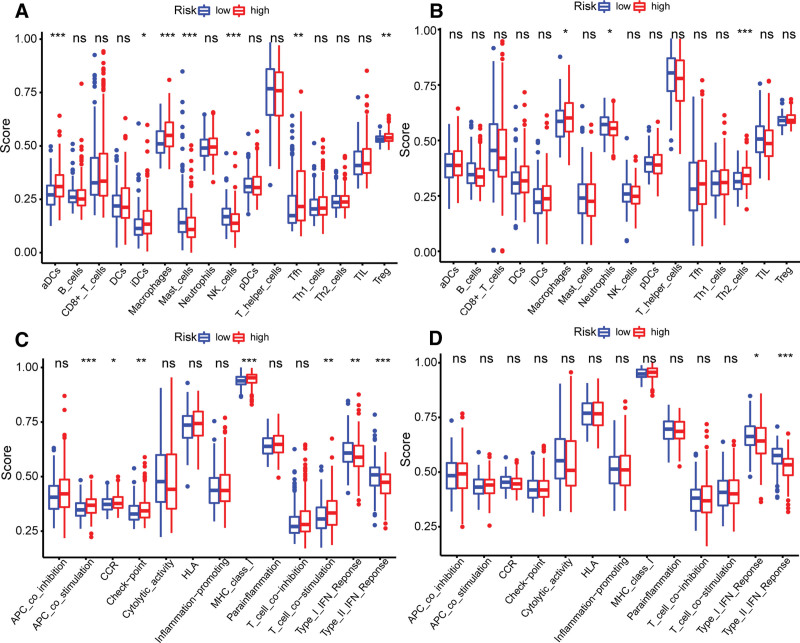
The ssGSEA analysis of immune cells and immune pathways. The ssGSEA analysis of 16 kinds of immune cells of low- and high-risk groups in the (A) TCGA cohort and the (B) ICGC cohort, and the ssGSEA analysis of 13 immune-related pathways in low- and high-risk groups in the (C) TCGA cohort and the (D) ICGC cohort. (NS: no significant difference, *P value < .05, **P value < .01, ***P value < .001; blue box: low risk group, red box: high risk group). ICGC = international cancer genome consortium, ssGSEA = single-sample gene set enrichment analysis.

Subsequently, we studied and understood the enrichment scores of 16 immune cells and the activities of 13 immune-related pathways in the low- and the high-risk groups of the TCGA and ICGC cohorts. The ssGSEA found that in the cohort of TCGA and ICGC, the infiltration level of macrophages in the high-risk group was higher than that in the low-risk group (Fig. 8A, B; *P* value < .05). In the TCGA and ICGC cohort, the activity of immune pathway type I IFN response, type II IFN response in the high-risk group was lower than that in the low-risk group (Fig. 8C, D; *P* value < .05).

## 4. Discussion

The researchers found some similarities between necroptosis and apoptosis and found that multiple cell death modes may coexist and interact with each other.^[14]^ However, the correlation between necroptosis-related genes and OS in patients with HCC is still largely unknown. In this study, we studied the mRNA levels of 68 known necroptosis-related genes in HCC and normal samples in the TCGA cohort. Through analysis, it was found that most of the necroptosis-related genes (72%) were differentially expressed in HCC and normal samples, 38 genes were upregulated in HCC samples and 11 genes were downregulated in HCC samples. The HCC patients were divided into 2 relatively independent clusters by consensus clustering analysis based on necroptosis-related DEGs in the TCGA cohort. Based on the analysis of the 2 clusters, it was found that there were significant differences in OS and clinical characteristics between the 2 clusters. Univariate Cox regression analysis showed that nearly half of the 49 DEGs genes were associated with OS in patients with HCC. These results significantly suggest the potential role of necroptosis-related genes in HCC and the possibility of using these necroptosis-related genes to establish prognostic models. We constructed a 10-gene signature model by LASSO Cox regression analysis to further evaluate the prognostic value of these necroptosis-related genes and then verified the good performance of the gene signature in external data sets. GO, KEGG, and ssGSEA analysis showed that DEGs between high- and low-risk groups were related to the activity of immune pathways and the level of immune cell infiltration, and many biological processes and pathways related to immunity had been enriched. We have reason to speculate that necroptosis can regulate the tumor immune microenvironment. The high-risk population in the TCGA and ICGC cohort had a higher proportion of macrophages. The researchers found that because macrophages and regulatory T cells play an important role in immune invasion, the increase of macrophage and regulatory T cells infiltration in the tumor microenvironment may be related to the poor prognosis of patients with liver cancer.^[15–17]^ In addition, most of the patients in the high-risk group had impaired antitumor immunity, including the decreased activity of type I IFN response and type II IFN response and decreased level of NK cell infiltration. Therefore, the weakening of antitumor immune function in the high-risk group may be one of the reasons for their poor prognosis.

Necroptosis is a new type of programmed cell death, which is closely related to the discovery and progression of tumors. Previous studies have shown that necroptosis plays a vital role in the migration and invasion of many types of cancers.^[18]^ In addition, necroptosis is considered to be a promising way to eliminate cancer cells.^[19]^ The necroptosis-related gene signature produced in this study can be used to predict the prognosis of patients with HCC. The gene signature consists of 10 necroptosis-related genes (CDKN2A, HAT1, HSP90AA1, IPMK, KLF9, MYCN, NDRG2, PLK1, SQSTM1, and TNFRSF21), most of which are upregulated in HCC tumor tissues and are associated with poor prognosis. Seven genes (CDKN2A, HAT1, HSP90AA1, MYCN, PLK1, SQSTM1, and TNFRSF21) were upregulated in HCC tissues and were high-risk genes (HR >1), while 2 genes (KLF9, NDRG2) were downregulated in HCC tissues and were low-risk genes (HR <1). CDKN2A is a tumor suppressor gene that encodes the p16INK4A protein. CDKN2A plays a negative regulatory role in cell cycle progression (G1-to-S phase transition) by interfering with the formation of the complex between CDK4/6 and cyclin D.^[20,21]^ HSP90AA1 is a multifunctional protein that participates in the assembly and nuclear transport of viral RNA polymerase subunits as a molecular chaperone and then forms a mature ternary polymerase complex.^[22]^ PLK1 is involved in a variety of mitotic events, including G2/M transition, centrosome maturation and separation, mitotic spindle formation, chromosome segregation, and cytokinesis.^[23–25]^ MYCN proto-oncogene bHLH transcription factor (MYCN) is a member of the MYC proto-oncogene family, which is closely related to the regulation of embryonal development.^[26]^ Oncogene MYCN has been shown to play a key regulatory role in many digestive system tumors.^[27]^ TNFRSF21 (also known as DR6) is a member of the extended tumor necrosis factor receptor superfamily, which can participate in caspase-mediated signal transduction.^[28]^

To sum up, we define a new prognostic model for HCC, which is composed of 10 necroptosis-related genes. Our research shows that necroptosis is closely related to HCC because most of the necroptosis-related genes are differentially expressed between normal tissues and HCC tissues. In addition, our risk score based on the risk model of 10 necroptosis-related genes is an independent risk factor for predicting prognosis in patients with TCGA and ICGC cohorts of HCC. Moreover, we found that necroptosis-related DEGs in high- and low-risk groups were closely related to tumor immunity. Our study provides a new gene signature for predicting the prognosis of patients with HCC and provides an important basis for the future study of the relationship between necroptosis-related genes and immunity in HCC.

## Author contributions

ZX contributed to the study design. XZ, JL, TW, MG and YX contributed to data collection. ZX and XZ performed statistical analysis and interpretation. ZX and YW drafted the manuscript. All authors contributed to the critical revision of the final manuscript.

## Acknowledgments

We are very grateful to Professor Yinlu Ding of the Department of Gastrointestinal Surgery, The Second Hospital, Cheeloo College of Medicine, Shandong University for its support for this project.

## Supplementary Material


